# The Effect of Block Ratio and Structure on the Thermosensitivity of Double and Triple Betaine Block Copolymers

**DOI:** 10.3390/molecules29020390

**Published:** 2024-01-12

**Authors:** Jongmin Lim, Hideki Matsuoka, Yusuke Kinoshita, Shin-ichi Yusa, Yoshiyuki Saruwatari

**Affiliations:** 1 Department of Polymer Chemistry, Kyoto University, Katsura, Nishikyo-ku, Kyoto 615-8510, Japan; lim.jongmin.m29@kyoto-u.jp; 2 Department of Applied Chemistry, Graduate School of Engineering, University of Hyogo, 2167 Shosha, Himeji 671-2280, Hyogo, Japan; yusuke.4021.dec@gmail.com (Y.K.); yusa@eng.u-hyogo.ac.jp (S.-i.Y.); 3 Osaka Organic Chemical Industry Ltd., 7-20 Azuchi-machi, 1chome, Chuo-ku, Osaka 541-0052, Japan; yoshiyuki_saruwatari@ooc.co.jp

**Keywords:** temperature-responsive polymers, polybetaines, polymer self-assembly, double hydrophilic block copolymers

## Abstract

AB-type and BAB-type betaine block copolymers composed of a carboxybetaine methacrylate and a sulfobetaine methacrylate, PGLBT-*b*-PSPE and PSPE-*b*-PGLBT-*b*-PSPE, respectively, were synthesized by one-pot RAFT polymerization. By optimizing the concentration of the monomer, initiator, and chain transfer agent, block extension with precise ratio control was enabled and a full conversion (~99%) of betaine monomers was achieved at each step. Two sets (total degree of polymerization: ~300 and ~600) of diblock copolymers having four different PGLBT:PSPE ratios were prepared to compare the influence of block ratio and molecular weight on the temperature-responsive behavior in aqueous solution. A turbidimetry and dynamic light scattering study revealed a shift to higher temperatures of the cloud point and micelle formation by increasing the ratio of PSPE, which exhibit upper critical solution temperature (UCST) behavior. PSPE-dominant diblocks created spherical micelles stabilized by PGLBT motifs, and the transition behavior diminished by decreasing the PSPE ratio. No particular change was found in the diblocks that had an identical AB ratio. This trend reappeared in the other set whose entire molecular weight approximately doubled, and each transition point was not recognizably impacted by the total molecular weight. For triblocks, the PSPE double ends provided a higher probability of interchain attractions and resulted in a more turbid solution at higher temperatures, compared to the diblocks which had similar block ratios and molecular weights. The intermediates assumed as network-like soft aggregates eventually rearranged to monodisperse flowerlike micelles. It is expected that the method for obtaining well-defined betaine block copolymers, as well as the relationship of the block ratio and the chain conformation to the temperature-responsive behavior, will be helpful for designing betaine-based polymeric applications.

## 1. Introduction

Polyzwitterions, containing a cationic group and an anionic group on their repeating unit, belong to a distinctive class of polyelectrolytes. This charge neutrality in an ionized state differentiates them from typical water-soluble polymers. The properties of polyzwitterions in aqueous media are not in common with those of general polyelectrolytes nor non-ionic polymers. Basically, no counterions are incorporated due to their intrinsic charge balance, unlike other typical polyelectrolytes. Hence in salt-free water, both charges in their repeating unit allow electrostatic interactions with adjacent motifs. Three interaction modes have been generally discussed: intra-mer, inter-mer, and inter-chain attraction or repulsion. Intra-mer interaction requires the bending of the spacer carbons on meeting the inner charged group with the outer charged group [1]. The energy calculation results of molecular mechanics on zwitterionic surfactants suggested that this pairing does not occur in aqueous media, and a more favorable interaction with water molecules rather than inner groups, large dipole moment, and steric hindrance could be the reason [2]. Delgado et al. proposed a “soliton-like” model of head-to-tail conformation with nearest neighbors in polysulfobetaines [3]. These intra- or interchain electrostatic interactions are thought to lead to a chain collapse of polyzwitterions and low solubility in salt-free water. Consequently, all attraction/repulsion forces can be diminished by additional salts which reduce the Debye length of the solution, representing the range of electrostatic interaction [4], and chain expansion occurs. This behavior is opposite to that of ordinary polyelectrolytes which become less soluble in saline solution by charge screening, referred as the anti-polyelectrolyte effect [4,5,6,7], as determined from the hydrodynamic radii [3,8,9], viscosity [10], or theoretical analysis [11].

Amongst polybetaines, polysulfobetaines explicitly show this behavior. Generally, polysulfobetaine chains are hard to dissolve in pure water and additional salt ions or heat must be applied to promote their solvation. The intra/interchain attraction between polysulfobetaines is so prevalent that the hydrophilic-to-hydrophobic transition occurs below a certain temperature [12] resulting in phase separation in water. Their upper critical solution temperature (UCST) is known to depend on the carbon spacer length which relates to hydrophilic/hydrophobic character as well as molecular weight and the chemical structure [13,14,15]. Incorporated polysulfobetaine motifs with relatively hydrophobic polymers [16,17,18], with crosslinked hydrogels [19,20], or on the surface of inorganic nanoparticles [21,22] provided a thermoresponsive character in the systems. In addition, the block copolymers with lower critical solution temperature (LCST)-type nonionic polymers [23,24,25,26,27] revealed “schizophrenic” micelle formation beyond their UCST and LCST. On the other hand, the stimuli-responsive character of polycarboxybetaines is triggered by pH instead of temperature. The charge neutrality of polycarboxybetaines composed of a weak acid (carboxylate) and a dimethylammonium becomes positive under acidic conditions (pKa~3) due to protonated carboxylate ends. In this state, the chains are no longer polyzwitterions and the solution behavior would be much closer to that of polycations. The charge-switching ability could be used to build active surfaces which can protect them from protein adhesion and kill and release bacteria [28,29,30]. Flux change upon the elution of aqueous solutions having various salt and pH conditions was demonstrated by the pH- and salt-responsive swelling behavior of polycarboxybetaine chains coated on the membranes [31]. These stimuli-responsive characteristics are more exploitable than non-ionic polymer counterparts because of the intrinsic antifouling ability of zwitterions [32,33,34,35]. Usually, hydrophilic polymers such as polyethylene glycol (PEG) or polyethylene glycol methacrylate (PEGMA) are coated on a bare surface to prevent protein adsorption, promoted by an electrical double layer of the liquid-substrate surface [36], hydrophobic interactions [37], and additional microbe growth [38]. The non-ionic hydrophilic polymers provide a hydration layer to ban initial adsorption, but a disrupted hydration layer upon contact with proteins made the film less effective than polyzwitterions, showing unperturbed water molecule ordering [39,40,41].

We have investigated a new combination of double hydrophilic betaine diblock copolymers composed of a carboxy- and a sulfobetaine methacrylate (PGLBT-*b*-PSPE), which is a stimuli-responsive double hydrophilic block copolymer (DHBC). Having temperature-responsive PSPE on the one hand and pH-responsive PGLBT on the other hand, the diblock copolymer could reversibly change their state in water from free chains to PSPE-centered micelles [42]. Recent discoveries represented similar self-assembly of this double hydrophilic block copolymer at higher content solutions [43] or in zwitterionic salt solutions [44]. However, the copolymerization of these two betaine monomers through conventional step-by-step RAFT polymerization always left a large extent of unreacted first block (PGLBT, used as macroCTA) [42,43,44], which is not easy to remove due to the similar chemical affinity with the final product. Although the product purified by precipitation yielded relatively pure diblock copolymers with narrow dispersity, the accurate prediction of the block ratio was not possible in this synthetic route. Hence, a detailed study of block ratio on the thermoresponsive characters was hardly available. The main reason was assumed to be the poor stability of the dithioester end group in water during synthesis and dialysis. By carefully modifying the type of CTA, ratio of CTA to initiator, and concentration of monomers under the consideration of theoretical chain livingness [45], PGLBT-*b*-PSPE but also PMPC-*b*-PSPE (incorporated with a phosphobetaine) and PSPE-*b*-PGLBT-*b*-PSPE triblocks were successfully synthesized by iterative polymerizations in our previous study [46]. Each polymerization step resulted in high conversion (~99%), which enabled chain extension without a purification step. In this study, the betaine block copolymers with different block ratios and a similar total degree of polymerization were obtained by the one-pot procedure to elucidate the influence of the PGLBT/PSPE ratio and the molecular weight on the temperature-responsive solution behavior.

## 2. Results and Discussions

### 2.1. The Preparation of PGLBT-b-PSPE with Controlled Block Ratios

**Scheme 1:**
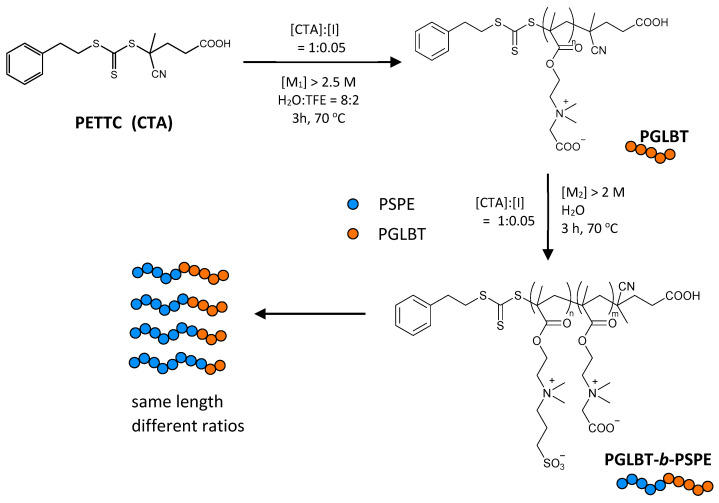
The synthetic route of PGLBT-*b*-PSPE via one-pot RAFT polymerization.

The AB-type diblock betaine copolymer PGLBT-*b*-PSPEs with various block ratios and total DP were prepared by the one-pot synthesis approach as done in the previous study [46]. The total target DP (DP_target_) was set to 300 and 600, and the aimed ratio of PGLBT:PSPE was varied from 1:1 to 1:5. The procedure of the synthesis and reaction conditions is described in Scheme 1, and the information for the obtained block copolymers is shown in Table 1. According to the fundamentals of RAFT polymerization, the activation–deactivation process does not affect the overall number of radicals, and radical sources (e.g., azoinitiator, redox initiator, or photoinitiator) must be incorporated to conduct polymerization. In the azoinitiator system, once a radical fragment provides radicals to monomers, chain propagation continues through the Z group and R group of the RAFT agent. The Z group determines the reactivity of C=S against radicals during the addition of the propagating radical and fragmentation step [47] while the R group reinitiates other monomers by acting as a homolytic leaving group [48]. As a result, two types of polymer chains are produced under a rough consideration: initiator-end chains without the Z group, and Z group-end chains. Therefore, the number of chains without the Z group, in other words “dead” chains, is governed by the initial amount of azoinitiator [49]. The Perrier group discussed the theoretical chain livingness calculated by comparing Z group-end chains to entire chains with consideration of the decomposition rate of azoinitiator, and successfully demonstrated multiblock copolymerization while keeping high chain livingness [45,49,50,51]. Consequently, the number of initiators introduced must be kept to a minimum as much as possible for suppressing initiator-end chain generation rather than CTA-end chains commencing further polymerization. The usage of less initiator, which necessarily lowers the polymerization rate is compensated for by increasing the concentrations of monomers [45], increasing reaction temperature to boost the decomposition rate of initiators, [51] using more reactive radical sources [45], and choosing good solvents for inducing polymerization acceleration [52]. 

In this study, VA-044 was used instead of other typical radical sources because of the higher decomposition rate (estimated to *k*_d_ = 4.2995 × 10^−4^ s^−1^ at 70 °C in the literature [45]) at the ratio of 0.05:1 = [VA-044]:[CTA], and monomer concentration [M] of 2.5–3 mol/L. Water is reported to increase the rate of propagation (*k*_p_) of vinyl monomers among other solvents [52], and is one of few solvents which can dissolve betaine monomers and polymers. Under the reaction conditions, the theoretical chain livingness (percentage of CTA-end chain) was lower than the ideal examples [45,50] because of the increased dosage of initiator ([CTA]:[I] was modified from 400 to 20) and lowered monomer concentration. This modification was inevitable due to our experimental conditions: the use of methacrylic monomers which have a much lower reactivity than acrylates, a different trithiocarbonate RAFT agent (PETTC) with the addition of organic solvent (TFE), and high target DPs particularly for synthesizing sufficiently long PSPE blocks having temperature-responsivity near room temperature. As discussed above, if the amount of initiator increases, the ratio of dead chain-end polymers increases. However, the initial radical concentration would become higher and promote more CTAs to participate in the polymerization. By regulating the concentration of monomers and initiators to keep the chain fidelity, the dead chain end percentages were kept ~5% at the first step and 10–14% at the second polymerization step. (details are shown in Tables S2 and S3 in Supporting Information) Also, the full conversion (~99%) of betaine monomers at each step of polymerization was successfully achieved at 3 h, and the result enabled consecutive betaine block extension without a pause regardless of the AB block ratio. TFE was added as a co-solvent with water only in the first polymerization step due to the enhancement of the insufficient solubility of PETTC in water, and it did not affect the polymerization rate significantly. 

**Figure 1:**
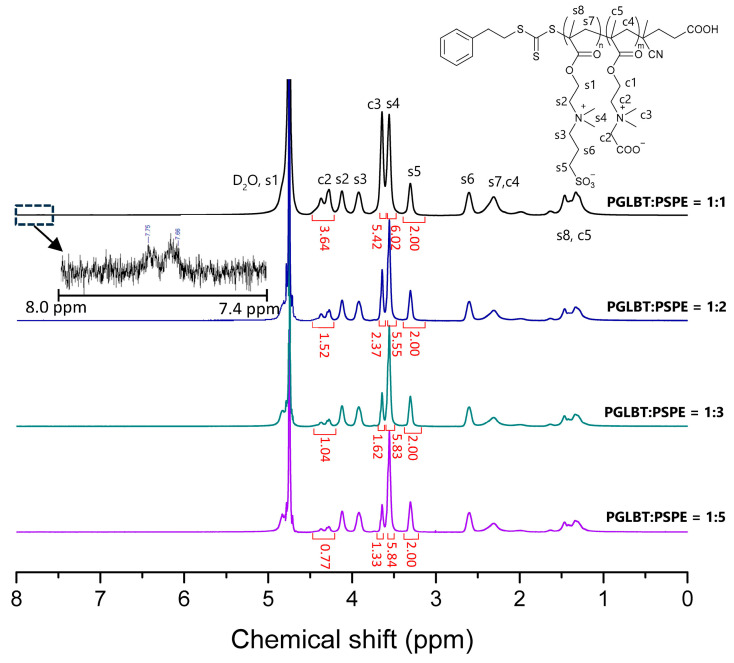
^1^H NMR spectra of four PGLBT-*b*-PSPEs having several ratios acquired in D_2_O at 60 °C. (DP_total_ = ~300) The inset shows an enlarged spectrum at 7.4–8.0 ppm.

**Figure 2:**
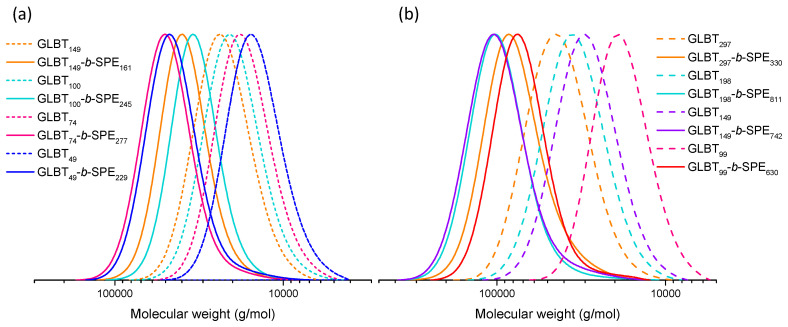
Molecular weight distributions of homo-PGLBTs (dashed lines) and PGLBT-*b*-PSPEs extended from the homo-PGLBTs. The total target DP was (**a**) 300 and (**b**) 600.

In the DP_total_ ~300 series, the obtained block copolymers had well-controlled ratios of PGLBT:PSPE as aimed for, with no residual PGLBT macroCTAs. The ratio and number-average molecular weight were calculated from ^1^H NMR spectra by comparing the signals of PSPE to those of PGLBT, measured at 60 °C to exclude zwitterionic attractions which reduce the intensity of PSPE signals. (Figure 1) The results of SEC analysis (Figure 2a) show clear curve shifts from lower molecular weight, representing homo-PGLBT, to higher molecular weight, indicating PGLBT-*b*-PSPE with narrow dispersities (<~1.2). In the case of DP_total_ = 600, some batches (GLBT_198_-*b*-SPE_811_, GLBT_149_-*b*-SPE_742_ and GLBT_99_-*b*-SPE_630_) resulted in incomplete chain extension in spite of the undetectable 1H NMR signals of SPE monomers in aliquots withdrawn even after 6 h of the reaction. Some residual homo-PGLBT was found on SEC chromatograms and removed by precipitation into MeOH (the SEC analysis results of before and after precipitation are displayed in Figure S1). Eventually, the ratios of polymers whose target ratio of PGLBT:PSPE = 1:2, 1:3 and 1:5 were estimated to be 1:4, 1:5 and 1:6. Nevertheless, the molecular weight distributions (MWDs) of copolymerized moieties were kept within the accepted range of well-performed RAFT polymerization (*Đ* < 1.3) as displayed in Table 1 and Figure 2b.

Since the residues did not occur on the DP_target_ = 300 batches, they may be attributed to the extremely increased viscosity which caused an inhomogeneous mixed state. Only GLBT_297_-*b*-SPE_330_ did not suffer this problem, presumably due to the identical target ratio per block that may ease the viscosity issue. The formation of residues was not thoroughly prevented by reducing the monomer concentration and increasing the amount of initiator at the second polymerization, which inevitably led to low chain livingness. However, it should be noted that the residual homo-PGLBT chains of the DP_target_ = 600 series were just a minor issue in comparison with the attempt to synthesize PGLBT-*b*-PSPE using a conventional step-by-step RAFT block copolymerization [42]. More than half of the macroCTA (homo-PGLBT) did not participate in SPE polymerization and the precise block ratio control was unachievable. The reaction time required was more than 10 hrs for each polymerization that resulted in lower conversions (~70%) due to the usage of a dithiobenzoate-type RAFT agent prone to retardation and more sensitive to hydrolysis [53]. Hence, three days of dialysis to wash out unreacted monomers after each polymerization was necessary, in contrast to the one-pot procedure which can extend multiple betaine motifs during 6–12 h with just a dialysis step at the end of the diblock copolymerization. The final yield of PGLBT-*b*-PSPE obtained using the previous method was about 40%, much lower than the yield (over 80%) in this study. 

**Figure 3:**
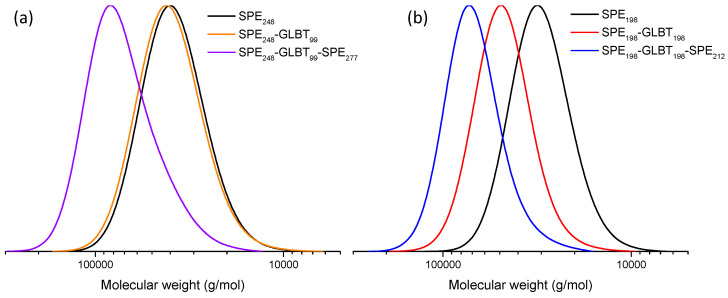
Molecular weight distributions for each of the three polymerization steps of the betaine triblock copolymer PSPE-*b*-PGLBT-*b*-PSPE (total DP~600): (**a**) SPE_248_-GLBT_99_-SPE_277_ (target ratio = 2.5:1:2.5) (**b**) SPE_198_-GLBT_198_-SPE_212_ (target ratio = 1:1:1).

For the synthesis of the BAB-type triblock, PSPE-*b*-PGLBT-*b*-PSPE, the chain extension was performed through the same one-pot procedure. Since we previously confirmed that the BAB triblock having the same overall AB composition (1:2:1) did not show significant temperature-responsive solution behavior [46], two PSPE-dominant copolymers were synthesized at the ratios of 2.5:1:2.5 and 1:1:1 to obtain the whole ratio of 5:1 and 2:1. Two consecutive chain growth steps were performed for 3 h, considered adequate to get full conversion (~99%) of the monomers, and any retardation or residues were not found on the SEC analysis results, as shown in Figure 3. Each DP was estimated by ^1^H NMR spectra as shown in Figure S2 and the results are in Table 2. The MWD curve at each step monomodally shifted to higher molecular weights in both cases. Note that the final product of the 2:5:1:2.5 batch showed the gradual tailing of MWD and this widened the dispersity. In the second step of the experiment, an additional initiator ([CTA]:[I] was modified from 20:1 to 12.5:1) was used to compensate the polymerization rate against decreased monomer concentration (2.5–3 mol/L to 1 mol/L) adjusted for workable viscosity. The theoretically estimated chain livingness was 88% at the second step and 84% at the final step, meanwhile the chain livingness of the 1:1:1 batch was 91% at the second step and 85% at the final step (Table S3). Hence, the tailing of SPE_248_-GLBT_99_-SPE_277_ is thought to originate from the increased portion of dead chains that are not able to reinitiate further polymerization.

### 2.2. The Influence of Block Ratio on the Solution Behavior of PGLBT-b-PSPE

**Figure 4:**
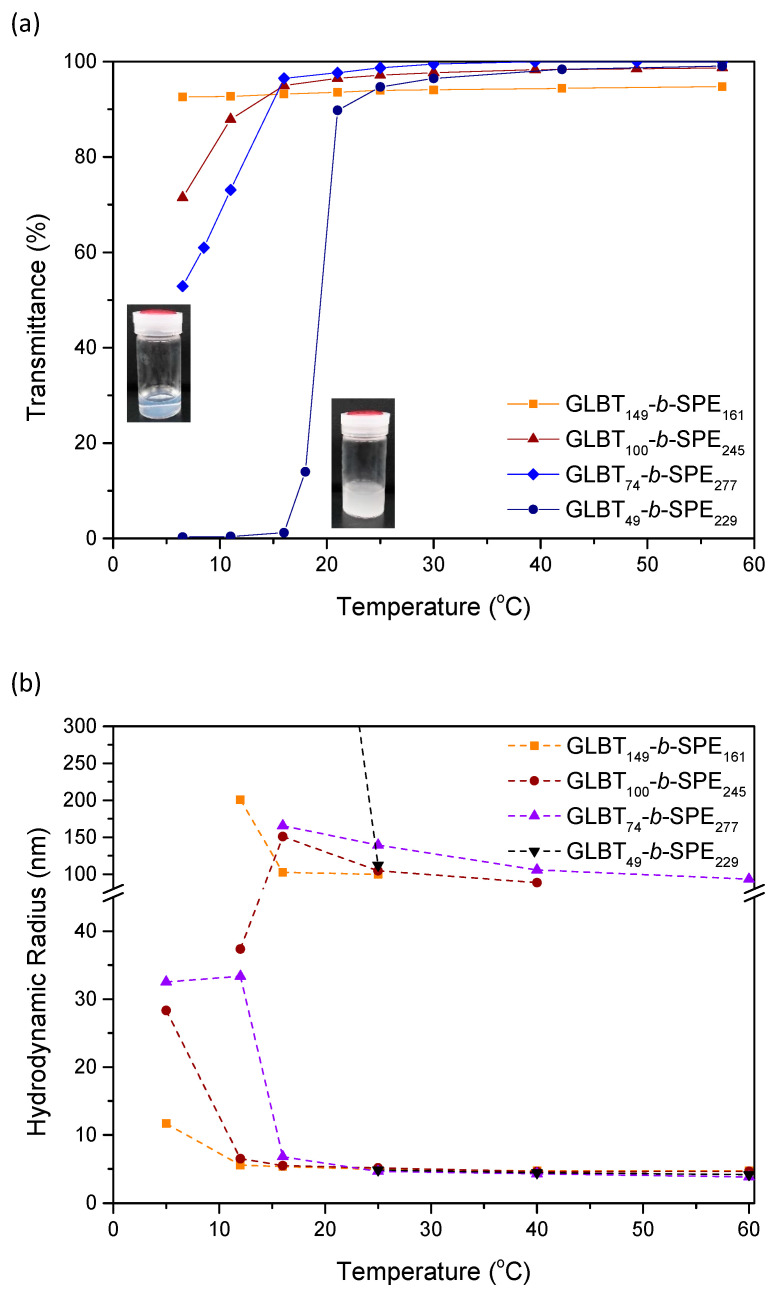
(**a**) Transmittance variations and (**b**) size variations against temperature in a series of PGLBT-*b*-PSPEs whose total target DP was 300. Insets of (**a**) are photos of GLBT_74_-*b*-SPE_277_ (**left**) and GLBT_49_-*b*-SPE_229_ (**right**) taken below 10 °C (sample concentration = 10 mg/mL).

Now the block ratio effect on the solution behavior of PGLBT-*b*-PSPE can be evaluated systematically by comparing the polymer samples having four different ratios and two different total target DPs. Firstly, the transmittance and size variation in the aqueous solutions of the DP_total_ ~300 series was investigated. Our studies hitherto revealed that sufficiently high portions of PSPE to PGLBT are required to render the UCST behavior in aqueous solutions above 0 °C, particularly for PSPE-centered micelle formation. Figure 4 and Table 3 show the transmittance increase/decrease and hydrodynamic radius change referring unimers (*R*_h_ < 10 nm), monodisperse micelles (*R*_h_ = 30–40 nm), or inter-chain assemblies (as intermediates, *R*_h_ > 100 nm) of respective sample solutions against temperature. As reported previously, no particular alteration of either transmittance or *R*_h_ happened on GLBT_149_-*b*-SPE_161_ solution because of a nearly identical unit number of both substances. It is thought that the temperature-independent PGLBT motifs intervened in associations among PSPE segments and the hydrophilicity of the entire chain was scarcely altered. Accordingly, the solution behavior did not show any clear differences even at the lowest end of the temperature range. The low ratio of larger objects (*R*_h_ > 100 nm, intensity-based relative weight = 10–50%), which are common in PGLBT-*b*-PSPEs that originate from the interchain attraction between PSPE pairs prior to phase separation, are accompanied by unimers at 5 °C.

**Figure 5:**
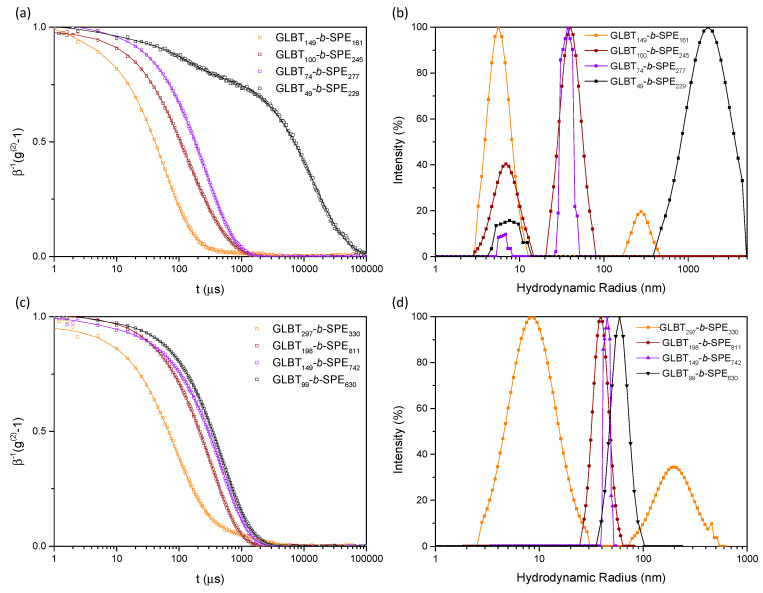
ACFs (**a**,**c**) and CONTIN analysis results (**b**,**d**) of PGLBT-*b*-PSPE aqueous solutions having different block ratios and total chain length. (**a**,**b**): DP_total_ = ~300, (**c**,**d**): DP_total_ = ~600. The ACFs and size distributions are obtained at 90° (scattering angle) and 12 °C. (sample concentration = 10 mg/mL).

More apparent thermoresponsivity appeared with the other polymer samples whose PSPE motif more than doubled that of PGLBT. The GLBT_100_-*b*-SPE_245_ solution showed a slight transmittance shift to ~70% while the unimers transformed to polymer micelles at 5 °C. GLBT_74_-*b*-SPE_277_ synthesized at the target ratio of 1:3 showed a more recognizable change, as expected. The transmittance dropped to ~50% at the end of the range, and the polymers in micellar form appeared below 12 °C. The clearest temperature-responsive behavior among the four was found in the GLBT_49_-*b*-SPE_229_ solution sample due to the sufficiently large portion of PSPE segment. The transmittance abruptly decreased to 0% as PSPE homopolymers under 20 °C, but intriguingly, micellar objects did not emerge unlike other typical PGLBT-*b*-PSPEs. The autocorrelation functions and analyzed *R*_h_ of four polymers at 12 °C in Figure 5a,b displayed different sensitivity against temperature: unimers and chain clusters (PGLBT:PSPE = 1:1), unimers and micelles (1:2), micelles (1:3), and size-grown aggregates (1:5). Among them, the GLBT_49_-*b*-SPE_229_ solution did not turn into a bluish translucent state attributed to Rayleigh scatterers, which are generally shown in micelle solutions, but rather a very turbid state in which white sediments settled to the bottom as time passed (see insets of Figure 4a). The hydrodynamic radius of this state was analyzed to be over 1000 nm with inhomogeneity via DLS (Figure 5a,b), nevertheless the high turbidity causing multiple scattering of the light source and angle-dependent scattering intensities made the precise analysis more complicated. In spite of GLBT_49_ segments providing hydrophilicity and repulsive forces against the opposite side of a chain, this phase separation repeatedly occurred under several heating and cooling cycles. 

Recalling our previous study [42], PGLBT-*b*-PSPE chains are in an intermediate state before rearranging monodisperse micelles in the cooling cycle. In this state, freely moving individual chains start to move close together due to zwitterionic attractions among PSPE but do not tightly bind together yet. These clustering objects are reflected as slow diffusive modes with fast diffusing unimers in DLS with subtle or gradual decreases of transmittance, but sedimentation does not occur. Both of the diffusive modes disappear and a new single-decaying ACF emerges, which indicates the reformation of monodisperse micelles (a typical example is shown at Figure S3). On the other hand, PSPE homopolymer chains cannot create polymer micelles owing to the lack of a permanent hydrophilic segment; thus, chain collapse and phase separation is inevitable. With GLBT_49_-*b*-SPE_229_ aqueous solution, it is thought that the PGLBT portion was insufficient to induce rearrangement from larger aggregates such as PSPE homopolymers, to monodisperse micelles. In addition, increasing the concentration (10 mg/mL to 40 mg/mL) of GLBT_49_-*b*-SPE_229_ triggered micellization at 25 °C (Figure S4), attributed to the shortened average distances of adjacent chains, but culminated in phase separation with additional cooling. Consequently, the distinctive phase behavior of GLBT_49_-*b*-SPE_229_ could mean that GLBT_49_ cannot maintain a stable layer covering the PSPE core, even if the micellar form once emerged. This phenomenon will be discussed further in the other samples of the DP_total_ ~600 series. 

**Figure 6:**
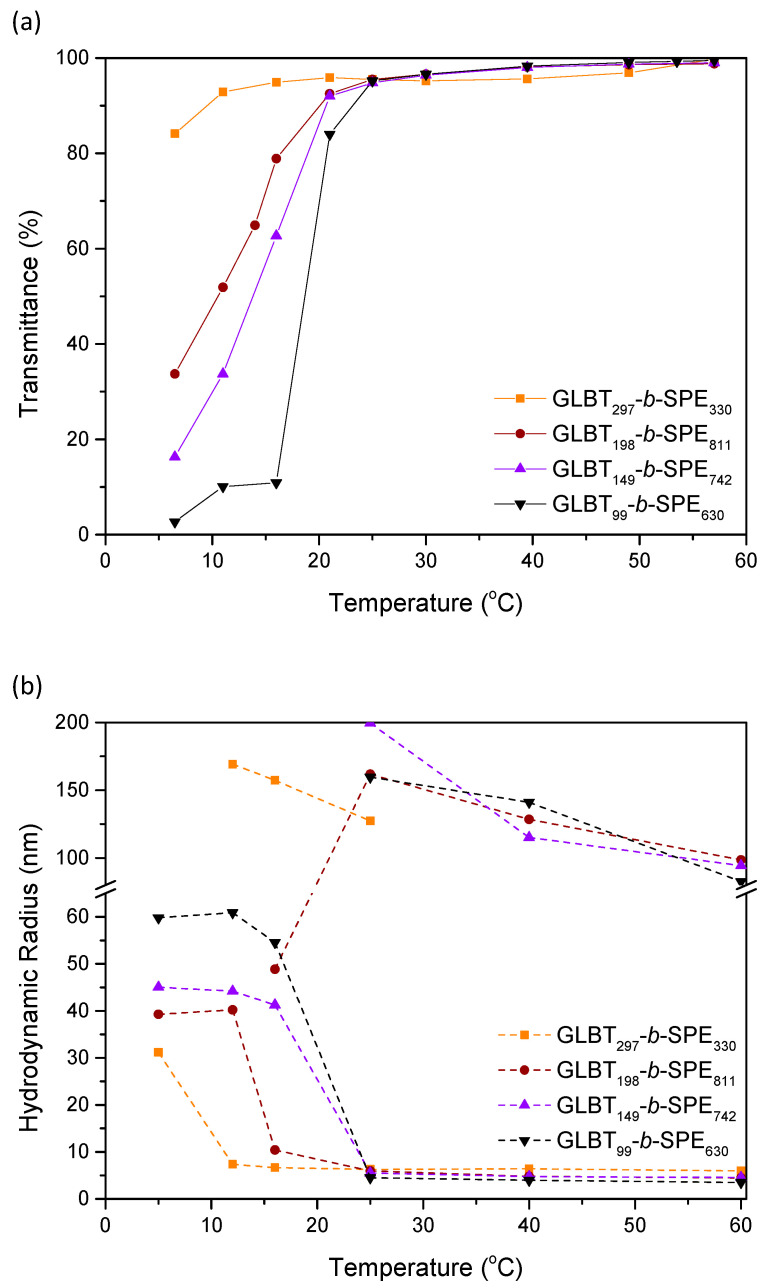
(**a**) Transmittance variations and (**b**) size variations against temperature in a series of PGLBT-b-PSPEs whose total target DP was 600 (sample concentration = 10 mg/mL).

The temperature-responsive solution behavior of PGLBT-*b*-PSPEs having twice the chain length and the same target block ratios is shown in Figure 6. Although their molecular weight approximately doubled, the polymer solutions did not show drastic differences in the trend and the transition points. Apparent thermoresponsive alterations did not occur in GLBT_297_-*b*-SPE_330_ (DP_target_ = 1:1) solution as in GLBT_149_-*b*-SPE_161_, but the chains took a micellar form (*R*_h_ = from 6 nm to 31 nm) at the low end of the temperature range with a slight transmittance decrease because of the slightly increased imbalance of both blocks. The other three polybetaine solutions showed clearer temperature-responsive behavior as their counterparts in the DP ~300 series; the unimer-to-micelle transition took place at higher temperatures, and GLBT_149_-*b*-SPE_742_ and GLBT_74_-*b*-SPE_277_ became micelles under 20 °C and under 15 °C, respectively. As shown in Figure 5c,d, three diblocks bearing more PSPE than PGLBT existed as monodisperse micelles at 12 °C whereas GLBT_297_-*b*-SPE_330_ was still in the intermediate state, represented as a bimodal ACF. It should be noted that the obtained PSPE/PGLBT block ratios of the DP_total_ ~600 diblock were higher than those of the DP_total_ ~300 diblock copolymers, so the transition shift to higher temperatures could be due to the increased portion of PSPE to PGLBT rather than the increase in total molecular weight. 

Note that GLBT_99_-*b*-SPE_630_, which is a counterpart of GLBT_49_-*b*-SPE_229_, did not undergo phase separation even at a higher PSPE to PGLBT ratio. Showing an abrupt transmittance shift around 20 °C as GLBT_49_-*b*-SPE_229_, GLBT_99_-*b*-SPE_630_ chains transformed to micelles (*R*_h_ = 60 nm) rather than immensely grown sediments, and the size of the micelles was maintained under additional cooling. In other words, no coalescence occurred after micellization. The intriguing difference between the two polymers suggests that there is a minimum DP of PGLBT for protection from the coalescence of adjacent PSPE cores and for stabilization of the core-shell type polymer micelles. 

**Table 3:** Cloud points and *R*_h_, *R*_g_ of PGLBT-*b*-PSPE in water. (DP_total_ = ~300 and ~600, sample concentration = 10 mg/mL) The *R*_h_ of unimers and micelles are based on the measurement at 60 °C and 12 °C, respectively.

	Cloud Point (°C)	*R*_h_ (Unimers) (nm)	*R*_h_ (Micelles) (nm)	PDI ^b^(Micelles)$\left(\mu_{2}/{\bar{\Gamma }}^{2}\right)$	*R*_g_ (Micelles) (nm)	*R_g_/R_h_*
GLBT_149_-*b*-SPE_161_	-	4.7	-	-	-	-
GLBT_100_-*b*-SPE_245_	-	4.6	28.3	0.12	41.7	1.47
GLBT_74_-*b*-SPE_277_	~5	3.8	33.4	0.13	28.1	0.84
GLBT_49_-*b*-SPE_229_	19.4	4.2	(phase separation)	-	-	-
GLBT_297_-*b*-SPE_330_	-	6.0	31.2	0.26	53.4	1.45
GLBT_198_-*b*-SPE_811_	8.4	4.5	40.2	0.15	33.9 ^a^	0.71 ^a^
GLBT_149_-*b*-SPE_742_	14.7	4.6	44.2	0.11	40.2 ^a^	0.78 ^a^
GLBT_99_-*b*-SPE_630_	18.7	3.5	60.9	0.16	60.4	0.99

^a^ The concentration was diluted to 5 mg/mL. ^b^ Obtained via cumulant analysis.

**Figure 7:**
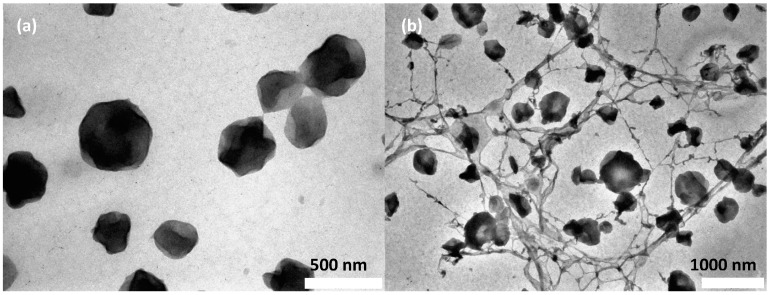
TEM images of GLBT_99_-*b*-SPE_630_ obtained from the solution in the micelle state: (**a**) spherical particles and (**b**) spherical objects with stringlike objects (conc. = 10 mg/mL).

The TEM images of the GLBT_99_-*b*-SPE_630_ solution revealed that the shape of the self-assembled polymer nanoparticles is spherical as shown in Figure 7a, and it corresponded to the *R*_g_/*R*_h_ (Table 3 and Figure S5). However, some anisotropic objects like strings or thin sheets were also found (Figure 7b). It should be noted that the solution cooled under the transition temperature was drop-cast on a TEM grid, then it was dried under an ambient temperature at which GLBT_99_-*b*-SPE_630_ existed in the intermediate state. Indeed, additional merging/disassociation could not be ruled out. The shape factor *R*_g_/*R*_h_ values were mainly 0.7–1.0 for the diblocks whose block ratio [PGLBT]:[PSPE] was over 1:2, and this indicates that the structure of the particles at low temperatures is spherical (*R*_g_/*R*_h_ = 0.775) [54,55]. The shape was also confirmed via the AFM measurement of a polymer solution-coated surface prepared at room temperature (Figure S7). In the additional measurement of the GLBT_99_-*b*-SPE_630_ sample whose concentration was diluted by half (5 mg/mL), strings and thin sheets were predominant (Figure S8) and the *R*_h_ of the polymer objects extraordinarily increased over 100 nm under 12 °C. The angle-dependent scattering intensity corroborates the anisotropically self-assembled state of the diluted sample. Since any extraordinary self-assembly was not found from the other PGLBT-*b*-PSPE samples diluted to 5 mg/mL, this block ratio and the chain length might yield the unexpected transformation when the distance between chains increased.

### 2.3. The Solution Behavior of the BAB Triblock PSPE-b-PGLBT-b-PSPE 

**Figure 8:**
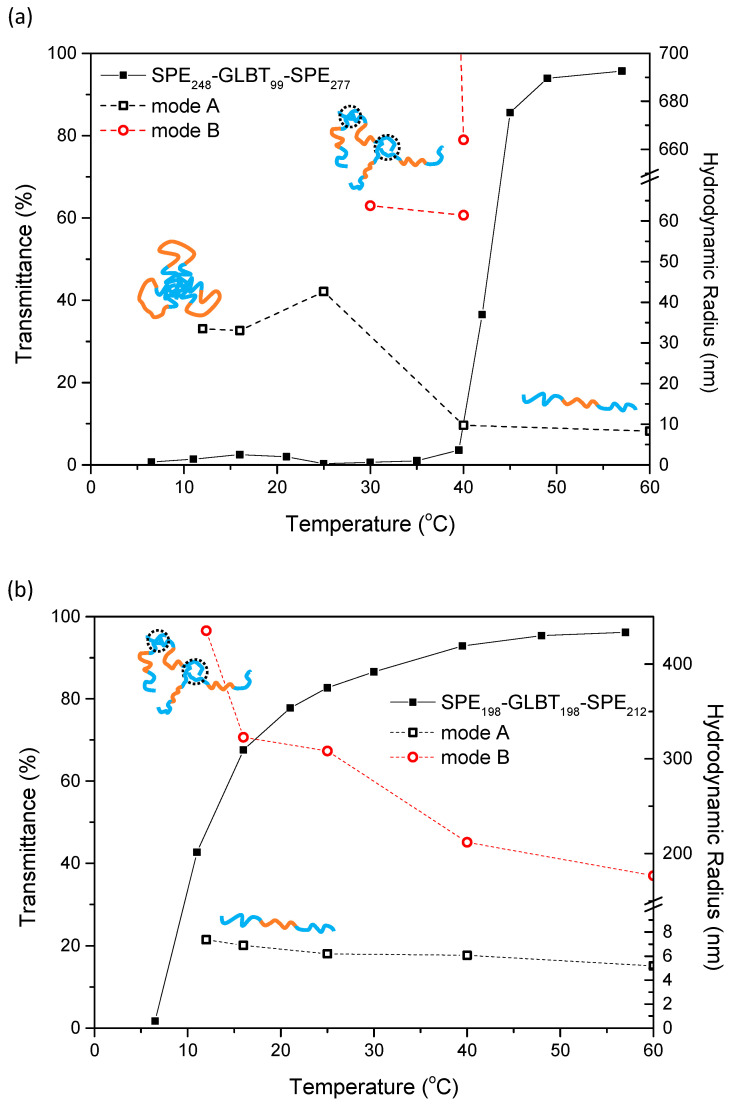
Variations in transmittance (■) and *R*_h_ (open symbols) against temperature for the triblock betaine copolymers (**a**) SPE_248_-GLBT_99_-SPE_277_ and (**b**) SPE_198_-GLBT_198_-SPE_212_ (sample concentration = 10 mg/mL). Inset figures represent the triblock chain state in water. (Blue: PSPE, orange: PGLBT).

**Figure 9:**
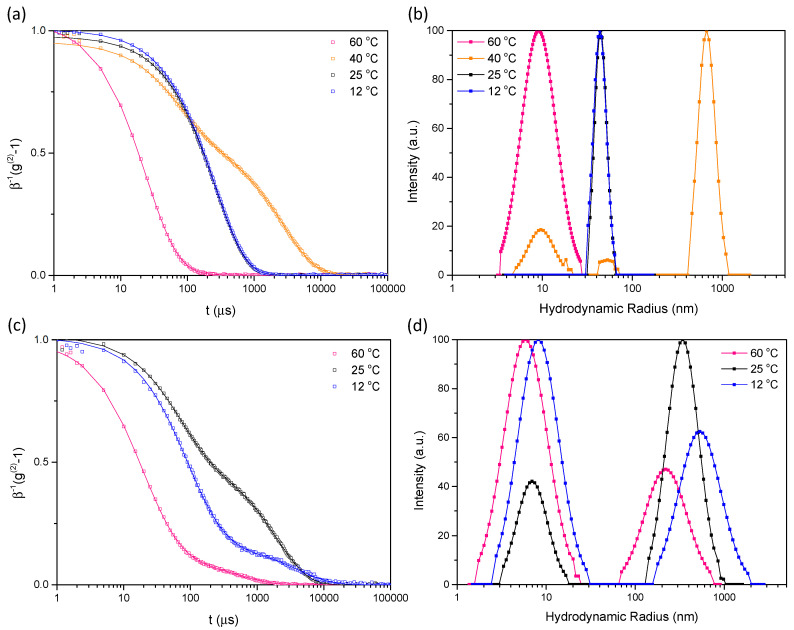
ACFs (**a**,**c**) and CONTIN analysis results (**b**,**d**) of two triblock PSPE-PGLBT-PSPE aqueous solutions. (**a**,**b**): SPE_248_-GLBT_99_-SPE_277_ and (**c**,**d**): SPE_198_-GLBT_198_-SPE_212_. The ACFs and size distributions were obtained at 90° (scattering angle) and 12 °C (sample concentration = 10 mg/mL).

The two AB-type triblock copolymers SPE_248_-GLBT_99_-SPE_277_ and SPE_198_-GLBT_198_-SPE_212_ in aqueous solution were investigated to determine the influences of the polymer structure on the thermoresponsive characters. BAB-type triblocks in which B blocks are able to associate with each other and A blocks are inert and provide unchanging water affinity may have B-centered particles (closed association) or a network formation connected by B blocks (bridging, open associations) [56]. For PSPE-PGLBT-PSPE, thermoresponsive zwitterionic attractions among PSPE would induce PSPE-centered particles having PGLBT outer loops (flower micelles) or loosely formed clusters consisting of associated PSPE nodes. The variations in transmittance and hydrodynamic radius against temperature are shown in Figure 8. The transmittance of SPE_248_-GLBT_99_-SPE_277_ aqueous solution rapidly dropped to a few % around 40 °C while triple diffusive modes existed, then became a hazy state until 25 °C. The turbid solution turned into a translucent state at 25 °C where a monomodal ACF representing monodisperse micelles (*R*_h_ = 43 nm) started to emerge, and the state was kept at lower temperatures as shown in Figure 9a,b that display ACFs and size distributions at several temperatures. The starkest contrast to the diblock of a similar block composition (GLBT_99_-*b*-SPE_630_) is extreme fuzziness below the cloud point which might be related to predominant slow modes at around 700 nm (at 40 °C) and over ~1900 nm (at 30 °C). These slow diffusive objects in the middle range of temperature are thought to be networks in which PSPE motifs are bound to other PSPEs of adjacent chains by bridging. However, similar to diblocks, the state transformed under 25 °C, as depicted in Figure 9a,b, showing a clear transition from a bimodal fast-slow decaying ACF to a unimodal decaying ACF. Once monodisperse particles (micelles) emerged, they continued to be present below the temperature. Therefore, the network-like objects are assumed not to be in a thermodynamically favored state under a certain temperature. To reduce the enthalpic penalty caused by decreasing temperature, close associations of zwitterionic pairing are thought to be preferred to reduce interfacial tension. Hence, the pairings rearrange to a flowerlike micellar form, which may be the more stable formation at much lower temperatures. The shape factor of the monodisperse particles (*R*_g_/*R*_h_ = 0.87) (Table 4 and Figure S6) suggests that the structure of PSPE-PGLBT-PSPE is spherical.

This behavior was expected to be repeated in the SPE_198_-GLBT_198_-SPE_212_ solution, but the thermoresponsive features appeared weaker and the chains did not reach the micelle state completely due to the reduced ratio of PSPE to PGLBT. However, compared to the AB-type diblock GLBT_198_-*b*-SPE_811_, the cloud point of the BAB-type was higher even with the lower PSPE balance (2:1 to 4:1), and the *R*_h_ of slow mode in the intermediate region was larger than that of the AB-type. While the transmittance gradually decreased until 15 °C, a slow diffusive mode (network-like assemblies) emerged then the relative percent of the slow mode exceeded that of unimers at 25 °C (Figure 9c,d). It is thought that the double-ended PSPE motifs which increase the probability of paring between adjacent PSPE motifs of different chains may attributed to the higher temperature sensitivity, but the PSPE/PGLBT ratio = ~2 is still not enough to form a stable micellar form as the diblock copolymer sample.

**Table 4:** Cloud points and *R*_h_, R_g_ of PSPE-*b*-PGLBT-*b*-PSPE in water (sample concentration = 10 mg/mL).

	Cloud Point (°C)	*R*_h_ (Unimer) (nm)	*R*_h_ (Micelles) (nm)	PDI ^b^ (Micelles) $\left(\mu_{2}/{\bar{\Gamma }}^{2}\right)$	*R*_g_ (Micelles) (nm)	*R_g_/R_h_*
SPE_248_-GLBT_99_-SPE_277_	42.8	8.31	43	0.14	57.6 ^a^	0.87 ^a^
SPE_198_-GLBT_198_-SPE_212_	12.5	5.20	-	-	-	-

^a^ The sample was diluted to conc. = 2.5 mg/mL. ^b^ obtained by cumulant analysis

## 3. Experimental Section

### 3.1. Materials

2-((2-(Methacryloyloxy)ethyl)dimethylammonio)acetate (carboxybetaine methacrylate, GLBT) and 3-((2-(methacryloyloxy)ethyl)dimethylammonio)propane-1-sulfonate (sulfobetaine methacrylate, SPE, which is often mentioned as SBMA in other reports) were kindly donated by Osaka Organic Chemical Industry LTD (Osaka, Japan) and used as received. 4-Cyano-4-(2-phenylethanesulfanylthiocarbonyl)sulfanylpentanoic acid (PETTC) was synthesized according to the literature [57] and used as a chain transfer agent (CTA). A radical initiator 2,2′-azobis[2-(2-imidazolin-2-yl)propane]dihydrochloride (VA-044) was purchased from Wako Chemicals (Osaka, Japan) and used as received. Ultrapure water (minimum resistivity ~18.2 MΩ cm) obtained via a Milli-Q system was used for the synthesis, dialysis, and preparation of polymer solutions. 2,2,2-trifluroethanol (TFE) was purchased from Nacalai Tesque and used as received. Dialysis was performed through regenerated cellulose membranes (MWCO 3500 and 15,000) to remove residues.

### 3.2. The Synthesis of PGLBT-b-PSPE via One-Pot RAFT Polymerization

GLBT, PETTC, and initiator were transferred into a rubber septum-sealed glass vial with a magnetic stirrer and dissolved into the mixture of water and TFE (8:2, *v*/*v*). The homogeneous solution was degassed with 15 min of argon bubbling, then immersed in an oil bath thermostated to 70 °C. The reaction was quenched in an ice bath after full consummation of GLBT monomers was identified using ^1^H nuclear magnetic resonance (NMR) analysis of aliquots. For the second polymerization, SPE, additional initiator and water were put into the vial and homogeneously dissolved. The required amount of SPE and initiator were determined based on the theoretical number-based molecular weight of PGLBTs. The reaction was started after 15 min of argon purging in the same manner, and terminated after the full conversion of SPE monomers, then the final product was purified via excess dialysis against Milli-Q water. The lyophilized product yielded a yellow powder. Generally, the reaction time was 3 h and extended to 6 h for the batches of DP_target_ = 600. Detailed reaction conditions are listed in Tables S1–S3 in the supporting information.

### 3.3. Polymer Characterization

- ^1^H NMR spectroscopy

The spectra of synthesized polymers that dissolved in deuterated water (D_2_O) were acquired using a 400 MHz JEOL JNM-AL400 spectrometer (JEOL, Tokyo, Japan). A minimum of 64 scans were recorded for each sample.

- Size exclusion chromatography (SEC)

The molecular weight distribution was determined using a column (SB-804 HQ, Shodex) and a refractive index detector (RI-830, JASCO, Japan) operating under an aqueous condition. A buffer solution (0.5 M CH_3_COOH and 0.3 M of Na_2_SO_4_) was used for elution at a flow rate of 0.5 mL/min as the mobile phase. The number-average molecular weight (*M*_n_) and dispersity (*M*_w_/*M*_n_, denoted as *Đ*) were determined via the calibration curve (third order fitting) obtained through five poly(2-vinylpyridine) standards. (*M*_n_: 5500, 10,300, 37,000, 78,500, 142,000 g/mol, Sigma-Aldrich).

- Turbidimetry

The transmittance variation in the block copolymer aqueous solutions (10 mg/mL) was recorded along a temperature range (from 60 °C to ~5 °C) by a UV-VIS spectrometer (Hitachi U-3310 spectrophotometer). A quartz cell with a light path of 10 mm was used, and the temperature was controlled via a water circulator appended to the cell holder. A transmittance of 200–600 nm was scanned at each temperature after 5 min of stabilization and values at 400 nm were taken for reporting. Each solution was gently heated to reach a transparent solution state then filtered through a syringe filter unit (pore size: 0.2 μm, mdi) prior to measurement.

- Light scattering

Dynamic light scattering (DLS) was carried out to determine the hydrodynamic radii of the polymer objects in aqueous solution (10 mg/mL). Sample cells were immersed in a temperature-controlled goniometer (BI-200SM, Brookhaven Instruments, New York, NY, USA) equipped with a 15 mW He-Ne laser (wavelength λ = 632.8 nm, index matching fluid = decahydronaphtalene). The field autocorrelation functions were obtained via a BI-DS2 photomultiplier tube with a correlator (TurboCorr, Brookhaven Instruments) at four scattering angles (60°, 75°, 90°, and 105°). Static light scattering (SLS) was carried out in a single concentration (10 mg/mL or 5 mg/mL) to measure the *R*_g_ of micellar objects. All of the experiments were started after no scattering intensity fluctuation was confirmed. The temperature varied from high (60 °C) to low (5 °C) in order to exclude kinetically trapped chain complexes. The details of the light scattering measurement are described in the supporting information.

- Transmission electron microscopy (TEM)

TEM observations were performed using a JEOL (Tokyo, Japan) JEM-2100 with an accelerating voltage of 160 KV. All samples for TEM observation were prepared by placing one drop of the aqueous solution on a copper grid coated with thin films of Formvar and carbon. Excess water was blotted using filter paper. The samples were stained with sodium phosphotungstate aqueous solution (0.2 wt%), dropped and blotted, and dried under vacuum for 1 day. TEM samples which should be prepared with cooling were prepared in a cooling device, Funakoshi (Tokyo, Japan) Cryoporter CS-80C.

### 3.4. The Determination of Monomer Conversion and Theoretical Molecular Weight

The monomer conversion was calculated from ^1^H NMR spectra using the following equation as described in the literature [51]:

(1) $$p=\frac{{\left[M\right]}_{0}-{\left[M\right]}_{t}}{{\left[M\right]}_{0}}=1-\frac{{\left[M\right]}_{t}}{{\left[M\right]}_{0}}=1-\frac{\frac{\int^{}I_{5.7-6.2\;\mathrm{ppm}}}{\int^{}I_{CTA}}}{{\mathrm{DP}}_{\mathrm{target}}}$$

where [M]_0_ and [M]_t_ are the monomer concentrations at the initial and elapsed time t, ∫ *I*_5.7–6.2 ppm_/∫ *I*_CTA_ is the corrected proton ratio of the unreacted monomer to the CTA (7.2–7.5 ppm) at the end of the chain. DP_target_ is the targeted number-average degree of polymerization. Each conversion was also determined by the following equation:

(2) $$p=\frac{\int^{}I_{p}}{\int^{}I_{p}+\int^{}I_{m}}$$

where ∫ *I_p_* is the corrected integral value of polymer peaks (2.5–2.7, 3.2–3.4, 3.4–3.6, 3.8–4.0, and 4.0–4.2 ppm for PSPE, 3.6–3.7 and 4.2–4.5 ppm for PGLBT) and ∫ *I_m_* is for vinyl protons of the monomer (at 5.7–6.2 ppm). The theoretically predicted molecular weight ($M_{n}^{\mathrm{theo}}$) was determined using the conversion values:

(3) $$M_{n}^{\mathrm{theo}}=p\cdot {\mathrm{DP}}_{\mathrm{target}}\cdot M_{M}+M_{\mathrm{CTA}},\;\;{\mathrm{DP}}_{\mathrm{target}}=\frac{\left[M\right]}{\left[\mathrm{CTA}\right]}$$

where M_M_ and M_CTA_ are molecular weight of the monomer and the CTA, respectively.

## 4. Conclusions

The AB-type and BAB-type of carboxybetaine-*block*-sulfobetaine methacrylate block copolymers were obtained via one-pot aqueous RAFT polymerization with good control of the block ratio. For AB-type PGLBT-*b*-PSPEs, the aim was to have AB ratios of 1:1 to 1:5, and the cloud point of the unimer-to-micelle transition rose directly with increasing portion of PSPE. The unimer-to-micelle transition via temperature decrease was monitored via light scattering and demonstrated monodisperse micelles whose hydrodynamic radius was 30–60 nm. The static/dynamic light scattering analysis results corresponded with the TEM/AFM images. Insufficient PGLBT segment length led to phase separation rather than micelle formation under the critical temperature. However, there was no meaningful change in the solution behavior depending on total DP as found in the other series prepared with the same AB ratios. Thus, it is proposed that the effect of the entire chain length is not as significant as the block ratio on the temperature-responsive characteristics. For BAB triblock PSPE-PGLBT-PSPEs, the responses against temperature were more sensitive in comparison with diblocks having similar block ratios and chain lengths. The solutions became turbid at higher temperatures while the chains existed as unimers and slow diffusive objects a few folds larger than those of the equivalent diblocks, which are assumed to be in a network-like structure via pairing between adjacent PSPE segments. The triblocks in this state eventually reformed monodisperse particles at much lower temperatures, and it is thought to be flowerlike micelles of PGLBT loops on the outside and the PSPE core. We envision the elucidation of whole betaine block copolymers, covering from the synthetic procedure for well-defined di- or triblock copolymers to the crucial factors affecting the temperature-responsive behavior, might be useful for developing temperature-responsive soft materials which require a high antifouling property.The betaine-based polymeric applications such as stimuli-responsive nanocarriers for drug-delivery systems or active surfaces are expected.

## Figures and Tables

**Table 1:** Summary of the double betaine block copolymers synthesized in the study.

	*M*_n_^theo^ (g/mol)	*M*_n_^SEC^ (g/mol)	*Đ* (*M*_w_/*M*_n_)
GLBT_149_-*b*-SPE_161_	77,400	36,400	1.12
GLBT_100_-*b*-SPE_245_	90,300	43,800	1.17
GLBT_74_-*b*-SPE_277_	93,600	45,100	1.14
GLBT_49_-*b*-SPE_229_	74,900	41,700	1.15
GLBT_297_-*b*-SPE_330_	156,400	71,300	1.20
GLBT_198_-*b*-SPE_811_	269,500	89,100	1.18
GLBT_149_-*b*-SPE_742_	239,700	88,300	1.21
GLBT_99_-*b*-SPE_630_	197,600	66,500	1.16

**Table 2:** Summary of the BAB-type betaine triblock copolymers synthesized in the study.

	*M*_n_^theo^ (g/mol)	*M*_n_^SEC^ (g/mol)	*Đ* (*M*_w_/*M*_n_)
SPE_248_-GLBT_99_-SPE_277_	168,300	66,300	1.20
SPE_198_-GLBT_198_-SPE_212_	157,500	64,500	1.13

## Data Availability

Data are contained within the article and supplementary materials.

## Supplementary Materials

The following supporting information can be downloaded at: https://www.mdpi.com/article/10.3390/molecules29020390/s1.
