# Supplementary material for: The Effect of Block Ratio and Structure on the Thermosensitivity of Double and Triple Betaine Block Copolymers
Source: Molecules. 2024 Jan 12;29(2):390. doi: 10.3390/molecules29020390 (PMC10820771; doi:10.3390/molecules29020390)
Supplement: Supplementary file 1 [file molecules-29-00390-s001.zip › molecules-2669098-supplementary.pdf]

Supporting Information

Effect of Block Ratio and Structure on the Thermosensitivity of Double and Triple Betaine Block Copolymers

Jongmin Lim[§], *Hideki Matsuoka*[§]*, *Yusuke Kinoshita*[†], *Shin-ichi Yusa*[†], *and Yoshiyuki Saruwatari*[‡]

§ Department of Polymer Chemistry, Kyoto University, Katsura, Nishikyo-ku, Kyoto 615-8510, Japan

† Department of Applied Chemistry, Graduate School of Engineering, University of Hyogo, 2167 Shosha, Himeji, Hyogo 671-2280, Japan

‡ Osaka Organic Chemical Industry Ltd., 7-20 Azuchi-machi, 1chome, Chuo-ku, Osaka 541-0052, Japan

Table S1. Experimental conditions for the synthesis of PGLBT-*b*-PSPEs via RAFT polymerization in water at 70 °C (sum of DP_{target}: 300)

Sample	$GLBT_{149}$-*b*-SPE_{161}		$GLBT_{100}$-*b*-SPE_{245}		$GLBT_{74}$-*b*-SPE_{277}		$GLBT_{49}$-*b*-SPE_{229}	
**Steps**	**1**	**2**	**1**	**2**	**1**	**2**	**1**	**2**
Monomer	GLBT	SPE	GLBT	SPE	GLBT	SPE	GLBT	SPE
DP_{target}	150	150	100	200	75	225	50	250
$m_{monomer}$ (g)	1.427	1.851	0.951	2.469	0.713	2.814	0.476	3.086
m_{CTA} (g)	0.015		0.015		0.015		0.015	
$m_{VA\text{-}044}$ (g)	0.7×10^{-3}	0.7×10^{-3}	0.7×10^{-3}	0.7×10^{-3}	0.7×10^{-3}	0.7×10^{-3}	0.7×10^{-3}	0.7×10^{-3}
Reaction time (h)	3	3	3	3	3	3	3	3
$V_{solvent}$ (mL)[a]	2.2	2.7	1.7	4.2	1.3	4	0.75	4.25
$[M]_0$ (mol/L)	3.01	2.45	2.60	2.10	2.55	2.52	2.95	2.60
$[CTA]_0/[I]_0$	20	20	20	20	20	20	20	20
Cumulative L (%)	95.3	90.8	95.3	90.8	95.3	90.8	95.3	90.8

[a] is the sum of the volume of the solvent added in the first and the next step

Table S2. Experimental conditions for the synthesis of PGLBT-*b*-PSPEs via RAFT polymerization in water at 70 °C (sum of DP_{target}: 600)

**Sample**	**$GLBT_{297}$-*b*-SPE_{330}**		**$GLBT_{198}$-*b*-SPE_{811}**		**$GLBT_{149}$-*b*-SPE_{742}**		**$GLBT_{99}$-*b*-SPE_{630}**	
**Steps**	**1**	**2**	**1**	**2**	**1**	**2**	**1**	**2**
Monomer	GLBT	SPE	GLBT	SPE	GLBT	SPE	GLBT	SPE
DP_{target}	300	300	200	400	150	450	100	500
$m_{monomer}$ (g)	1.902	2.493	1.268	3.292	0.951	3.703	0.634	3.086
m_{CTA} (g)	0.01		0.01		0.01		0.01	
$m_{VA\text{-}044}$ (g)	0.5 x 10^{-3}	0.7 x 10^{-3}	0.5 x 10^{-3}	1.0 x 10^{-3}	0.5 x 10^{-3}	1.0 x 10^{-3}	0.5 x 10^{-3}	1.0 x 10^{-3}
Reaction time (h)	6	6	6	6	6	6	6	6
$V_{solvent}$ (mL)[a]	2.80	3.50	1.70	4.60	1.20	4.50	1.00	7.00
$[M]_0$ (mol/L)	3.16	2.55	3.46	2.56	3.68	2.95	2.95	2.12
$[CTA]_0/[I]_0$	20	14	20	10	20	10	20	10
Cumulative L (%)	95.3	90.8	95.3	86.6	95.3	86.6	95.3	86.7

Table S3. Experimental conditions for preparation of BAB-type PSPE-PGLBT-PSPEs via RAFT polymerization in water at 70 °C (sum of DP_{target}: 600)

**Sample**	**SPE_{198}-$GLBT_{198}$-SPE_{212}**			**SPE_{248}-$GLBT_{99}$-SPE_{277}**		
**Steps**	**1**	**2**	**3**	**1**	**2**	**3**
Monomer	SPE	GLBT	SPE	SPE	GLBT	SPE
DP_{target}	200	200	200	250	100	250
$m_{monomer}$ (g)	2.304	1.793	2.337	3.086	0.959	3.118
m_{CTA} (g)	0.014			0.015		
$m_{VA\text{-}044}$ (g)	0.7 x 10^{-3}	0.7 x 10^{-3}	0.9 x 10^{-3}	0.7 x 10^{-3}	1.2 x 10^{-3}	0.7 x 10^{-3}
Reaction time (h)	3	3	3	3	3	3
$V_{solvent}$ (mL)[a]	2.70	3.20	4.20	3.60	4.10	5.60
$[M]_0$ (mol/L)	3.05	2.60	2.00	3.07	1.09	1.99
$[CTA]_0/[I]_0$	20	20	14	20	12.5	20
Cumulative L (%)	95.3	90.8	84.9	95.3	88.3	84.1

Details of DLS and SLS analysis

The normalized intensity autocorrelation function $g^{(2)}(q,t)$ is measured by

$$g^{(2)}(q,t) = \frac{\langle I^*(q,0)I(q,t)\rangle}{\langle I(q,0)^2\rangle} \tag{S1}$$

Then, $g^{(2)}(q,t)$ is related to the normalized electric field autocorrelation function $g^{(1)}(q,t)$ by the Siegert relation (S1),

$$g^{(2)}(q,t) = 1 + \beta\left|g^{(1)}(q,t)\right|^2 \tag{S2}$$

where β is a coherence factor depending on the experimental conditions, and $g^{(1)}(q,t)$ is to be evaluated. For a polydisperse particle system, the $g^{(1)}(q,t)$s can be written as:

$$g^{(1)}(q,t) = \int_0^\infty G(\Gamma)\exp\left(-\Gamma t\right)d\Gamma \tag{S3}$$

where G(Γ) is the intensity-based normalized distribution of decay rates $\int G(\Gamma)d\Gamma = 1$ and able to be calculated through inverse Laplace transform. (CONTIN regularization was applied by a software, BIC Particle Explorer, Brookhaven Instruments) The translational diffusion coefficient $D_\mathrm{t} = \bar{\Gamma}/q^2$ was obtained from the slope of the decay rate $\bar{\Gamma}$ versus q^2. The scattering vector q is

$$q = \frac{4\pi n}{\lambda}\sin\left(\frac{\theta}{2}\right) \tag{S4}$$

where n is the refractive index of the water, λ is the wavelength of the incident laser (632.8 nm), and θ is the scattering angle. The hydrodynamic radius R_h was obtained by the Stokes-Einstein equation:

$$R_{\mathrm{h}} = \frac{k_B T}{6\pi\eta D_t} \tag{S5}$$

Where k_{B} is the Boltzmann constant, T is the absolute temperature, and η is the viscosity of solvent (water), respectively.

In the static light scattering (SLS) measurements, the scattering intensity were recorded over an angular range between 40° and 150° at 10° intervals. A partial Zimm equation (S5) of the inverse scattering intensity ($1/I_{\mathrm{ex}}$) versus the square of the scattering vector q^2 at a single concentration was used to determine the radius of gyration (R_{g}), as follows

$$\frac{1}{I_{ex}(q)} = C(1 + \frac{R_g^2 q^2}{3}) \tag{S6}$$

where C is the arbitrary constant, and $I_{\mathrm{ex}}(\mathrm{q})$ is the intensity of scattered light. Several points that were apparently discrepant from the overall results were exempted from the linear fitting.

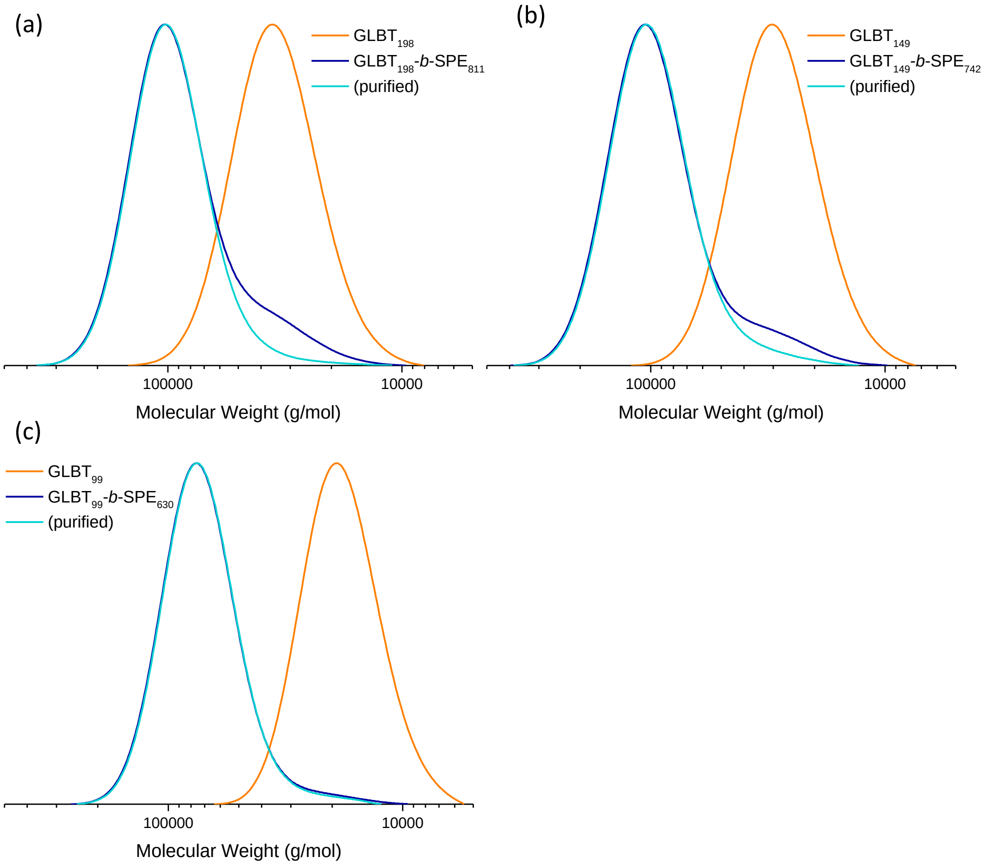

Figure S1. Molecular weight distributions of crude and purified PGLBT-*b*-PSPEs and PGLBTs as their macroCTAs (total DP_{target}: 600) (a) $GLBT_{198}$-*b*-SPE_{811} (b) $GLBT_{149}$-*b*-SPE_{742} (c) $GLBT_{99}$-*b*-SPE_{630}

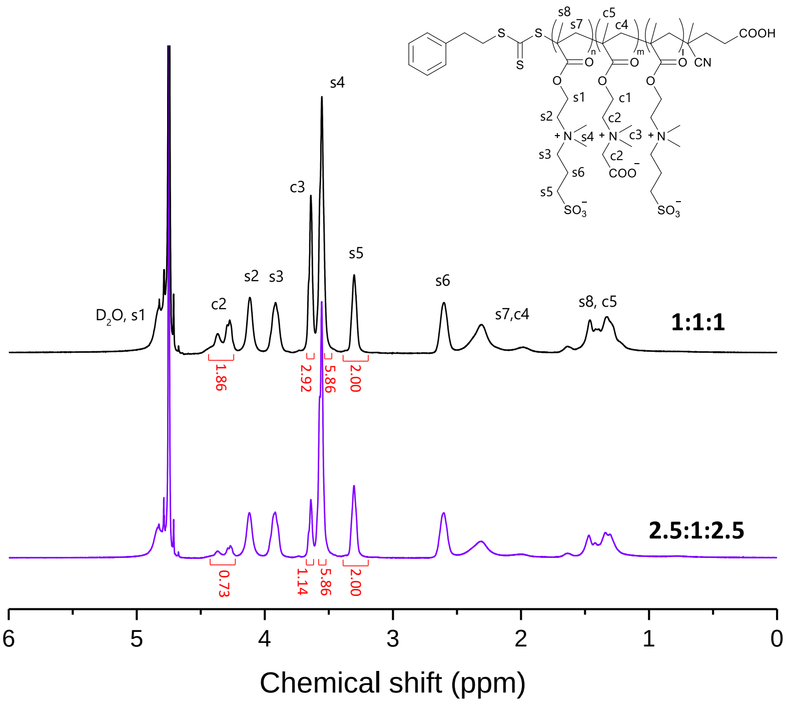

Figure S2. ^{1}H NMR spectra of two triblocks in D_2O at 60 °C. SPE_{198}-$GLBT_{198}$-SPE_{212} (target ratio = 1:1:1, black line) and SPE_{248}-$GLBT_{99}$-SPE_{277} (target ratio = 2.5:1:2.5, blue line)

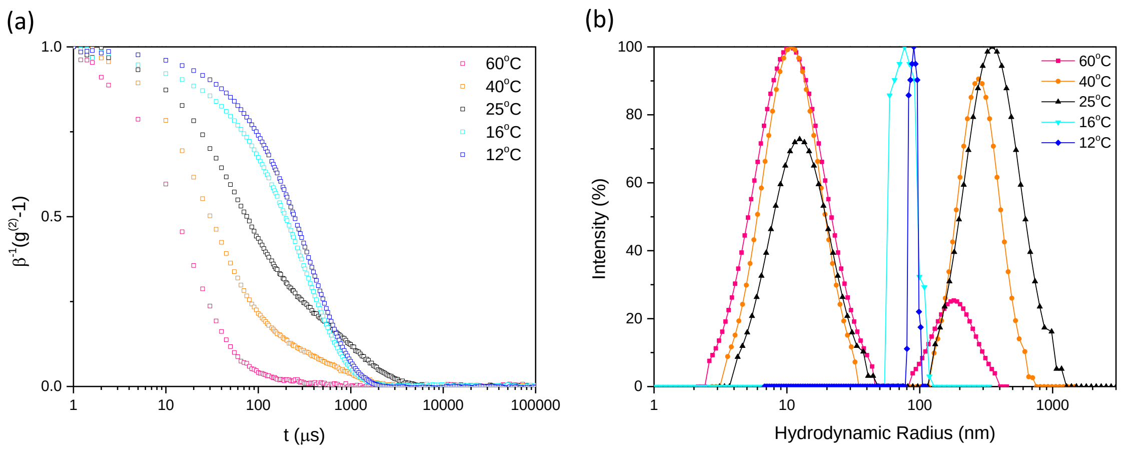

Figure S3. The autocorrelation functions of $GLBT_{149}$-*b*-SPE_{742} (a) and the size distributions (b) at 5 temperatures. (conc. = 10 mg/mL)

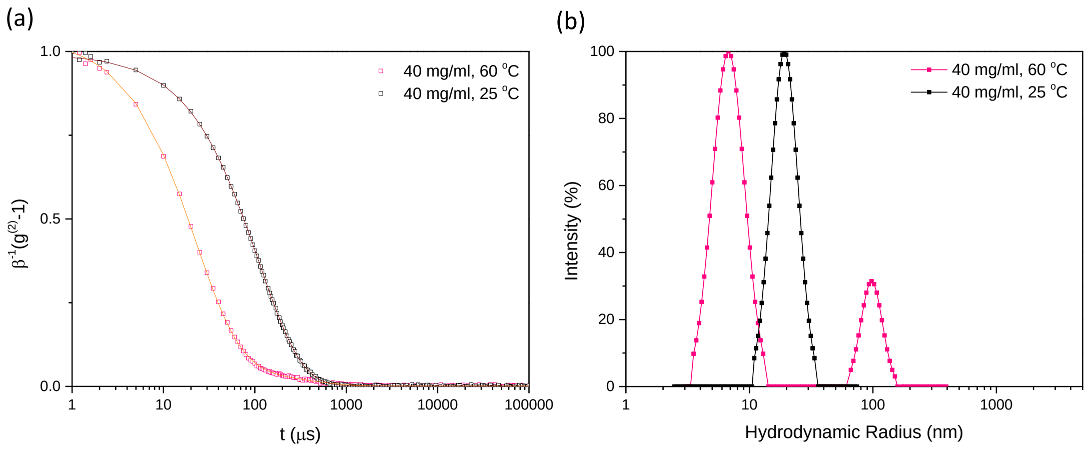

Figure S4. (a) ACFs and (b) the CONTIN analysis result of $GLBT_{49}$-*b*-SPE_{229} solution (conc. = 40 mg/ml) measured at 25 and 60 °C, respectively.

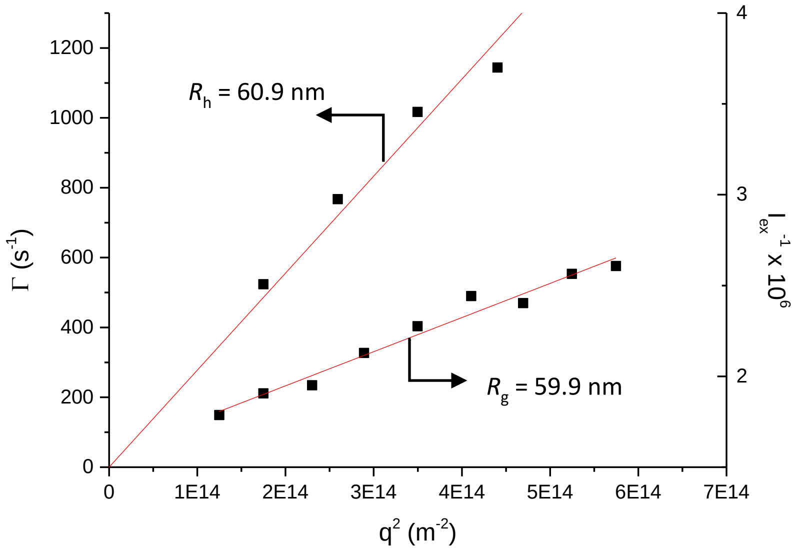

Figure S5. Angular dependent DLS and SLS measurements of (a) $GLBT_{99}$-*b*-SPE_{630} at 12 °C (conc. = 10 mg/mL)

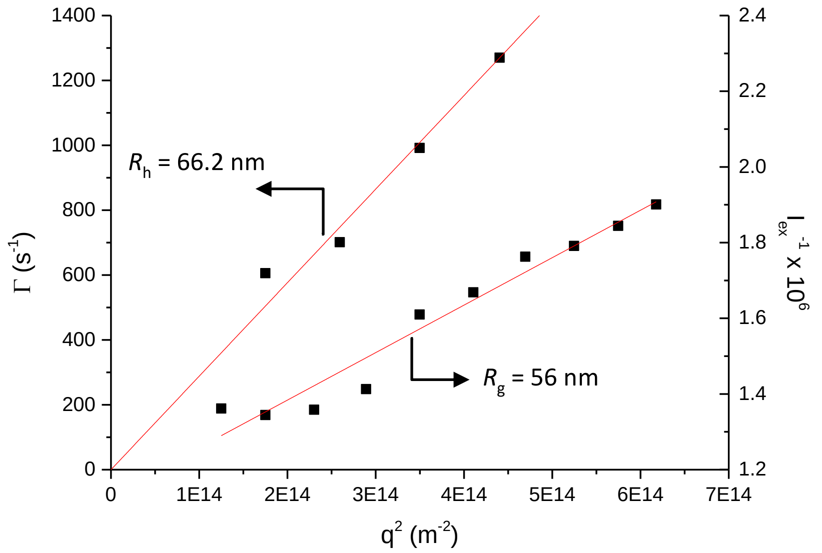

Figure S6. Angular dependent DLS and SLS measurements of (a) SPE_{248}-$GLBT_{99}$-SPE_{277} at 16 °C (conc. = 2.5 mg/mL)

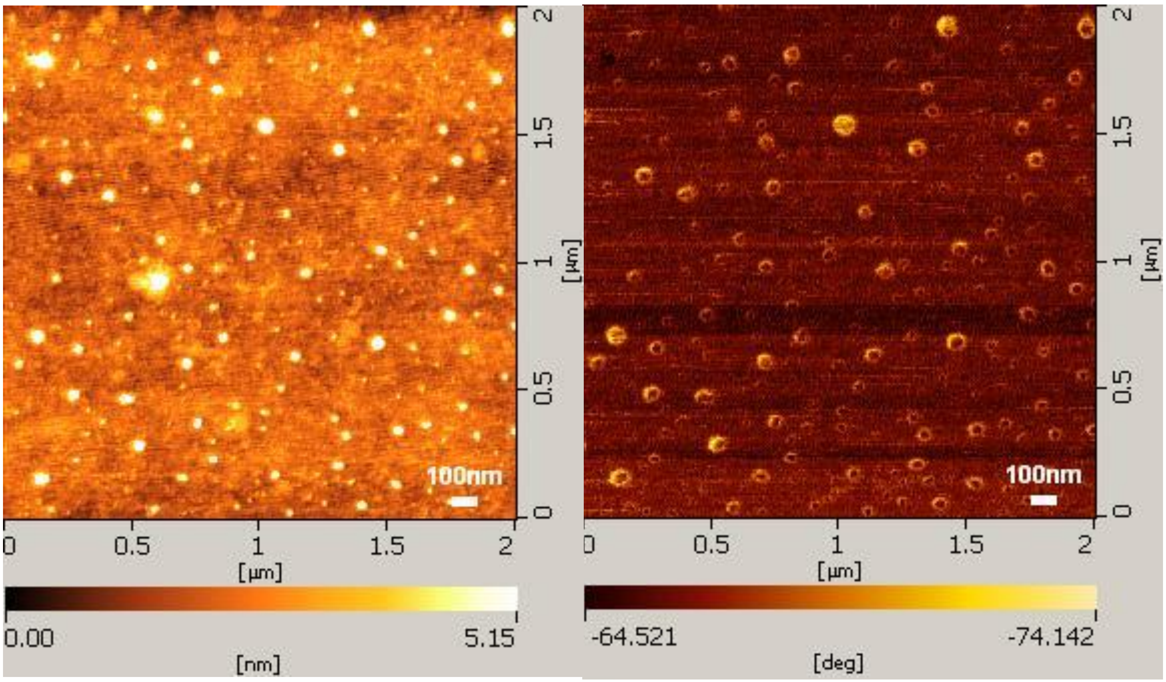

Figure S7. AFM image of $GLBT_{198}$-*b*-SPE_{811} (50 mg/mL) on a glass substrate (left: height mode, flattened, right: phase mode).

The AFM measurement was carried out under the following condition: A Seiko SPI3800 probe station an SPI300 unit system in dynamic force mode (non-contact mode) were operated with microcantilevers. (OMCL-AC240TS-C3, Olympus, typical resonance frequency: 70 kHz and typical spring constant: 1.7 N/m) For sample preparation, aqueous suspensions of colloidal PGLBT-*b*-PSPE particles was dropcast onto a microscope slide glass (IWAKI, Japan) and dried at room temperature more than three days.

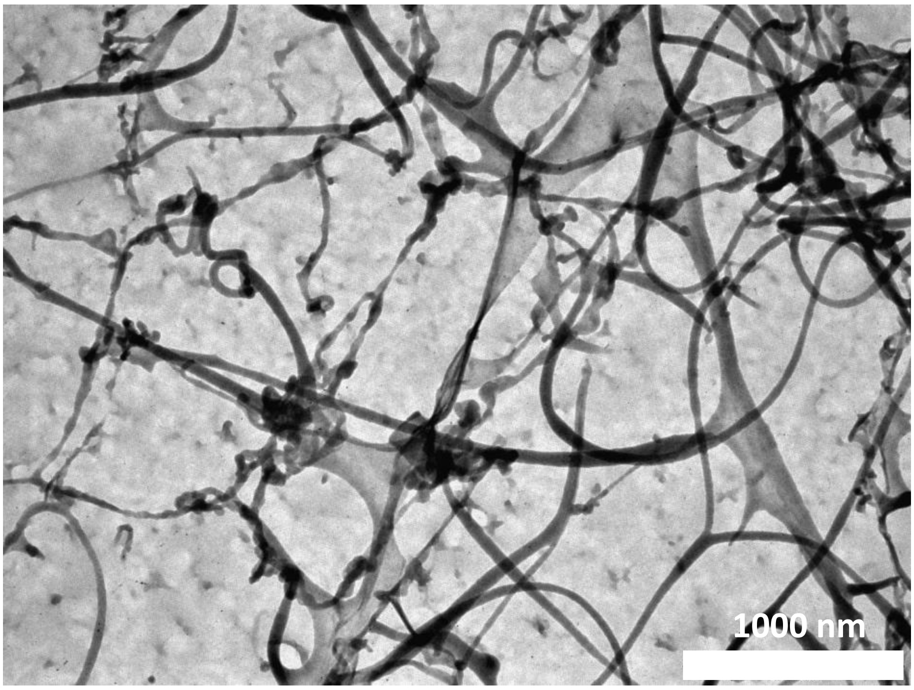

Figure S8. TEM image of $GLBT_{99}$-*b*-SPE_{630} obtained from the solution in the self-assembled state (conc. = 5 mg/mL)
